# Real-time delay-multiply-and-sum beamforming with coherence factor for *in vivo* clinical photoacoustic imaging of humans

**DOI:** 10.1016/j.pacs.2019.100136

**Published:** 2019-08-09

**Authors:** Seungwan Jeon, Eun-Yeong Park, Wonseok Choi, Ravi Managuli, Ki jong Lee, Chulhong Kim

**Affiliations:** a Department of Creative IT Engineering, Pohang University of Science and Technology (POSTECH), Pohang, 37673, Republic of Korea; b Department of Electrical Engineering, Pohang University of Science and Technology (POSTECH), Pohang, 37673, Republic of Korea; c Department of Bioengineering, University of Washington, Seattle, WA, 98195, USA; d Hitachi Medical Systems of America, Twinsburg, OH, 44087, USA; e Future IT Innovation Laboratory, Pohang University of Science and Technology (POSTECH), Pohang, 37673, Republic of Korea; f Departments of Creative IT Engineering, Mechanical Engineering, and Electrical Engineering, Pohang University of Science and Technology (POSTECH), Pohang, 37673, Republic of Korea

**Keywords:** Optoacoustic, Image reconstruction, Imeage enhancement, High frame rate

## Abstract

In the clinical photoacoustic (PA) imaging, ultrasound (US) array transducers are typically used to provide B-mode images in real-time. To form a B-mode image, delay-and-sum (DAS) beamforming algorithm is the most commonly used algorithm because of its ease of implementation. However, this algorithm suffers from low image resolution and low contrast drawbacks. To address this issue, delay-multiply-and-sum (DMAS) beamforming algorithm has been developed to provide enhanced image quality with higher contrast, and narrower main lobe compared but has limitations on the imaging speed for clinical applications. In this paper, we present an enhanced real-time DMAS algorithm with modified coherence factor (CF) for clinical PA imaging of humans *in vivo*. Our algorithm improves the lateral resolution and signal-to-noise ratio (SNR) of original DMAS beamformer by suppressing the background noise and side lobes using the coherence of received signals. We optimized the computations of the proposed DMAS with CF (DMAS-CF) to achieve real-time frame rate imaging on a graphics processing unit (GPU). To evaluate the proposed algorithm, we implemented DAS and DMAS with/without CF on a clinical US/PA imaging system and quantitatively assessed their processing speed and image quality. The processing time to reconstruct one B-mode image using DAS, DAS with CF (DAS-CF), DMAS, and DMAS-CF algorithms was 7.5, 7.6, 11.1, and 11.3 ms, respectively, all achieving the real-time imaging frame rate. In terms of the image quality, the proposed DMAS-CF algorithm improved the lateral resolution and SNR by 55.4% and 93.6 dB, respectively, compared to the DAS algorithm in the phantom imaging experiments. We believe the proposed DMAS-CF algorithm and its real-time implementation contributes significantly to the improvement of imaging quality of clinical US/PA imaging system.

## 1 Introduction

Photoacoustic imaging (PAI) is a medical imaging technique based on the photoacoustic (PA) effect that converts light energy into ultrasound (US) energy. Compared to other medical imaging techniques, PAI has several unique advantages. First, it can provide strong optical absorption contrasts with high ultrasonic spatial resolution in real-time [1]. Second, this medical imaging technique is safe for humans since it does not require contrast agents or ionizing radiation [2]. Third, it can provide functional information (*e.g.*, oxygen saturation) as well as morphological information by using multiple wavelengths [3,4]. Fourth, PA imaging has high compatibility with the US imaging modality, which is commonly used in routine clinical practice, because it shares the imaging source and the reconstruction methods [5,6]. PA and US images can be acquired simultaneously and used complementarily for medical diagnosis [[7], [8], [9]]. Thus, PAI has demonstrated the potential for image-based diagnosis of various diseases such as cancer [10,11], peripheral artery disease (PAD) [12], dermatitis [13], and arthritis [14].

As mentioned above, the PA signals are generated by the optical absorbers after absorbing the light, and the reconstructed PA images represent the initial distribution of the absorbers within the imaging area. Among the image reconstruction methods, the delay-and-sum (DAS) beamforming method is the most commonly used algorithm to reconstruct the images in both PA and US imaging [[15], [16], [17], [18], [19]]. The DAS sums the corresponding US signals by adjusting their time delays according to the distance between the source and the detectors [[20], [21], [22], [23]]. However, DAS has some drawbacks such as low resolution, low contrast, and strong side lobes resulting in artifacts. In 2015, Matrone et al. proposed a novel beamforming algorithm, called the delay-multiply-and-sum (DMAS) beamformer, to overcome the limitations of DAS in US imaging [24,25]. DMAS provides the enhanced image quality with higher contrast, narrower main lobes, and weaker side lobes than DAS.

Due to these advantages of DMAS, several researchers have adopted DMAS in PA imaging. Park et al. introduced a DMAS-based synthetic aperture focusing technique to PA microscopy in 2016 [26]. Alshaya et al. demonstrated the DMAS PA imaging with a linear array transducer and additionally introduced a subgroup DMAS method to improve the SNR and processing speed [27]. To improve the image quality of DMAS further, Mozaffarzadeh et al. proposed using double-stage DMAS operation [28], a minimum variance beamforming algorithm [29], or modified coherence factor [30]. Kirchner et al. developed a signed DMAS (sDMAS) algorithm to better preserve the important PA information in the low-frequency domain than the existing DMAS and accelerated the sDMAS through graphic processing unit (GPU) processing. Despite these advances, it has been challenging to utilize DMAS clinically due to the heavy computation complexity of *O*(*N^2^*) required to incorporate this algorithm into the clinical PA imaging system.

In this study, our goals are twofold. First, we present an enhanced DMAS algorithm with a modified coherence factor (CF) [31], to achieve better spatial resolution and signal-to-noise ratio (SNR) compared to the existing DMAS beamformer algorithm. Second, to optimize the computing requirements of this algorithm for integrating into a programmable clinical PA system [32] for real-time performance. Note that CF has also been applied to another advanced beamforming, called minimum-variance beamforming (MVBF) [33]. However, MVBF is not capable of real-time imaging because its inverse matrix computation is highly complex and difficult to optimize. On the other hand, the proposed DMAS-based beamforming can be practically implemented because the real-time beamforming is available through optimization. We compare the image quality of our proposed DMAS-CF against existing DMAS algorithm to show the superiority in the image quality using our algorithm. We also incorporate this algorithm into the clinical PA system for real-time evaluation *in vivo*. In the next few sections, we present our algorithm in greater detail along with real-time implementation into the clinical PA system. We also present a real-time *in vivo* evaluation of human forearm images and show the outstanding spatial resolution and SNR of our algorithm compared to other beamforming algorithms. Finally, we present a discussion and conclusion.

## 2 Method

### 2.1 Delay-and-sum beamforming and a coherence factor

PA images are commonly reconstructed by using DAS beamforming algorithm which is defined as follows:

(1) $$S_{DAS}\left(t\right)=\sum_{i=1}^{N}a_{i}s_{i}^{'}\left(t+\Delta t_{i}\right)=\sum_{i=1}^{N}s_{i}\left(t+\Delta t_{i}\right)$$

where *S_DAS_* is the output of the DAS beamformer, *N* is the receiving aperture size, $a_{i}$ is apodization coefficient, and *s_i_’*(*t+*Δ*t_i_*) is the signal detected by the *i*-th element with the corresponding time delay, Δ*t_i_*. However, this DAS beamformer has relatively poor lateral resolution due to the strong side lobes. A CF, a nonlinear weighting function, was introduced to overcome this problem of side lobes. The DAS is combined with CF (DAS-CF) as follows [20,31]:

(2) $$S_{DAS-CF}\left(t\right)=S_{DAS}\left(t\right)\times CF_{DAS}(t)$$

where

(3) $${CF}_{DAS}\left(t\right)=\frac{{\left(\sum_{i=1}^{N}s_{i}\left(t+\Delta t_{i}\right)\right)}^{2}}{N\sum_{i=1}^{N}{\left(s_{i}\left(t+\Delta t_{i}\right)\right)}^{2}}.$$

The CF can improve the SNR as well as the spatial resolution by effectively reducing the side lobes and noise levels.

### 2.2 Proposed delay-multiply-and-sum beamforming with a coherence factor

DMAS beamformer, a novel beamforming method, achieves enhanced contrast and lateral resolution compared to DAS and is defined as follows [24,26,34].

(4) $$S_{DMAS}\left(t\right)=\sum_{i=1}^{N-1}\sum_{j=i+1}^{N}\bar{\sqrt{s_{i}\left(t+\Delta t_{i}\right)}}\times \bar{\sqrt{s_{j}\left(t+\Delta t_{j}\right)}}$$

where

(5) $$\bar{\sqrt{a}}=\text{s}\text{g}\text{n}\left(a\right)\sqrt{\left|a\right|}.$$

Here, *S_DMAS_* is the output of the DMAS beamformer, and sgn(∙) is the signum. At first, the received signals, *s_i_*(*t*), are adjusted by the time delays, Δ*t_i_*, according to the distance between the source and the detectors as in DAS. Then, square roots are applied to the delayed signals while maintaining the respective signs as shown in Eq. (5). The signals were then combinatorially multiplied with each other and summed. Due to the combinatorial multiplication in Eq. (4), the center frequency, *f_0_*, of the original signals is shifted to DC and *2f_0_* in the output. Thus, the output needs to be filtered by a band-pass filter, centered at *2f_0_*, to extract the second harmonic components while removing the DC components.

Similar to Eq. (2), we combined the DMAS beamformer with the CF to reduce side lobes of DMAS further. The CF in DAS calculates the coherence of the *N* terms and reduces the intensity where the coherence is low. Similarly, the CF for DMAS calculates the coherence of the *N*(*N-*1)/2 terms in Eq. (4) as follows:

(6) $${CF}_{DMAS}\left(t\right)=\frac{{\left(\sum_{i=1}^{N-1}\sum_{j=i+1}^{N}\bar{\sqrt{s_{i}\left(t+\Delta t_{i}\right)}}\bar{\sqrt{s_{j}\left(t+\Delta t_{j}\right)}}\right)}^{2}}{\frac{N\left(N-1\right)}{2}\sum_{i=1}^{N-1}\sum_{j=i+1}^{N}{\left(\bar{\sqrt{s_{i}\left(t+\Delta t_{i}\right)}}\bar{\sqrt{s_{j}\left(t+\Delta t_{j}\right)}}\right)}^{2}}\;=\frac{{\left(\sum_{i=1}^{N-1}\sum_{j=i+1}^{N}\bar{\sqrt{s_{i}\left(t+\Delta t_{i}\right)}}\bar{\sqrt{s_{j}\left(t+\Delta t_{j}\right)}}\right)}^{2}}{\frac{N(N-1)}{2}\sum_{i=1}^{N-1}\sum_{j=i+1}^{N}\left|s_{i}\left(t+\Delta t_{i}\right)\right|\left|s_{j}\left(t+\Delta t_{j}\right)\right|}.$$

Therefore, the proposed DMAS-CF is defined as follow:

(7) $$S_{DMAS-CF}=S_{DMAS}\left(t\right)\times {CF}_{DMAS}\left(t\right)=\frac{{\left(\sum_{i=1}^{N-1}\sum_{j=i+1}^{N}\bar{\sqrt{s_{i}\left(t+\Delta t_{i}\right)}}\bar{\sqrt{s_{j}\left(t+\Delta t_{j}\right)}}\right)}^{3}}{\frac{N\left(N-1\right)}{2}\sum_{i=1}^{N-1}\sum_{j=i+1}^{N}\left|s_{i}\left(t+\Delta t_{i}\right)\right|\left|s_{j}\left(t+\Delta t_{j}\right)\right|}.$$

In Eq. (7), both the denominator and the numerator have the combinatorial multiplication operations. The combinatorial multiplication requires *N*(*N*-1)/2 multiplication operations, so about *N*(*N*-1) multiplications would be needed in DMAS-CF in total for each output of beamformer. This high computing complexity of *O*(*N^2^*) to generate each individual output makes it difficult to achieve real-time processing speed. For example, for a typical beamformer with *N* = 128 in PA imaging, the number of multiplication needed would be 16,390 for each pixel. This computing requirement is about 128 times more than the DAS algorithm, which is prohibitively compute-intensive. Thus, optimization is needed to reduce the computation burden, while maintaining the advantages of the algorithm.

To avoid the combinatorial multiplication, we reformed the DMAS-CF in Eq. (7) as follows:

(8) $$S_{DMAS-CF}=\frac{{\left[\left\{{\left(\sum_{i=1}^{N}\bar{\sqrt{s_{i}\left(t+\Delta t_{i}\right)}}\right)}^{2}-\sum_{i=1}^{N}{\left(\bar{\sqrt{s_{i}\left(t+\Delta t_{i}\right)}}\right)}^{2}\right\}/2\right]}^{3}}{\frac{N\left(N-1\right)}{2}\left[\left\{{\left(\sum_{i=1}^{N}\left|s_{i}\left(t+\Delta t_{i}\right)\right|\right)}^{2}-\sum_{i=1}^{N}{\left|s_{i}\left(t+\Delta t_{i}\right)\right|}^{2}\right\}/2\right]}\;=\frac{{\left[\left\{{\left(\sum_{i=1}^{N}\bar{\sqrt{s_{i}\left(t+\Delta t_{i}\right)}}\right)}^{2}-\sum_{i=1}^{N}\left|s_{i}\left(t+\Delta t_{i}\right)\right|\right\}\right]}^{3}}{2N\left(N-1\right)\left[\left\{{\left(\sum_{i=1}^{N}\left|s_{i}\left(t+\Delta t_{i}\right)\right|\right)}^{2}-\sum_{i=1}^{N}{\left|s_{i}\left(t+\Delta t_{i}\right)\right|}^{2}\right\}\right]}.$$

Note that the DMAS-CF in Eq. (8) generates mathematically the same result with the Eq. (7) because

(9) $$\sum_{i=1}^{N-1}\sum_{j=i+1}^{N}x_{i}x_{j}=\sum \left(\begin{array}{ccccc} 0 & x_{1}x_{2} & x_{1}x_{3} & \ldots & x_{1}x_{n}\\ 0 & 0 & x_{2}x_{3} & \ldots & x_{2}x_{n}\\ 0 & 0 & 0 & \ldots & \ldots \\ \ldots & \ldots & \ldots & \ldots & x_{n-1}x_{n}\\ 0 & 0 & 0 & \ldots & 0 \end{array}\right)=\frac{\sum XX^{T}-tr\left(XX^{T}\right)}{2}=\frac{{\left(\sum_{i=1}^{N}x_{i}\right)}^{2}-\sum_{i=1}^{N}x_{i}^{2}}{2}\text{w}\text{h}\text{e}\text{r}\text{e}\;X=\left(\begin{array}{c} x_{1}\\ x_{2}\\ \ldots \\ x_{n} \end{array}\right).$$

**Fig. 1:**
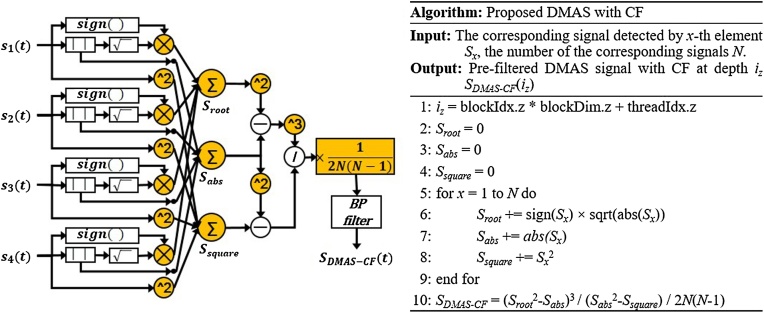
Diagram (left) and pseudocode (right) of DMAS-CF*.* DMAS, delay-multiply-and-sum; and CF, coherence factor.

This method requires another square operation, but no combinatorial multiplication is required (Fig. 1). As a result, the number of the multiplication operation in the DMAS-CF reduces significantly to only *2 N* per output and the complexity reduces from *O*(*N^2^*) to *O*(*N*). In Fig. 1, we show the pseudo-code to implement the proposed DMAS-CF algorithm.

### 2.3 Clinical Photoacoustic/Ultrasound system

**Fig. 2:**
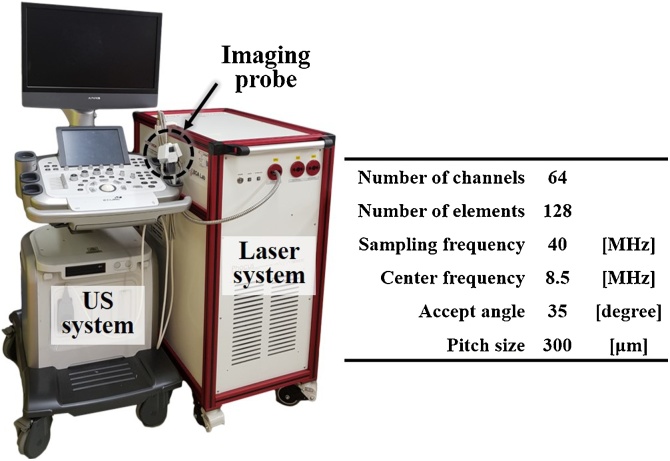
Configuration and parameters of the clinical PA/US imaging system. PA, photoacoustic; and the US, ultrasound.

Fig. 2 shows the clinical PA/US imaging system used for implementing the proposed DMAS-CF beamforming algorithm. The system was recently developed by Kim et al. using an FDA-cleared commercial research ultrasound system (ECUBE 12R, Alpinion Medical Systems, Republic of Korea) and a portable laser system (Phocus Mobile, OPOTEK Inc., USA) with the pulse repetition rate (PRF) of 10 Hz and the pulse energy of 10.1 mJ/cm^2^ [32,35]. We extracted 16-bit raw data from the system at a sampling rate of 40 MHz using a 128-element linear array transducer (8.5 MHz L3-12, Alpinion Medical Systems, Republic of Korea). The output of beamforming has 128 scanlines and 6 cm depth pixels. During the beamforming process, we applied a dynamic receiving aperture according to the transducer acceptance angle, and applied the apodization using the Hanning window. From the beamformed data, one B-mode image was generated through typical PA image processing steps including band-pass filtering, envelope detection, logarithmic compression, and scan conversion.

### 2.4 Phantom imaging

We imaged a phantom composed of 8 black nylon threads to compare the lateral resolutions of the PA images reconstructed by the DAS, DAS-CF, DMAS, and the proposed DMAS-CF beamforming algorithms. The nylon threads in the phantom had a diameter of 0.10 mm and were placed vertically at 10-mm intervals. We positioned the nylon thread phantom in water instead of in a tissue-mimicking phantom [36] to evaluate the beamforming methods under the relatively ideal condition. The SNRs of each line target were also measured for each algorithm. In the phantom imaging experiment, PA images were acquired with an excitation wavelength of 850 nm.

### 2.5 *In vivo* human imaging

We recruited three healthy volunteers to obtain *in vivo* images of the human forearm. All imaging procedures followed the protocol approved by POSTECH's Institutional Review Board. Informed consents were received from all volunteers after explaining the protocol. PA images were acquired in two different scanning modes: the handheld scanning mode and the stationary scanning mode. In the handheld scanning mode, a water bag was placed between the forearm and the probe, and commercial US gel was applied to match the acoustic impedance. Note that the water bag not only provides the axial distance (3 cm) that the laser beams can reach the center of the imaging plane from the side of the imaging probe (elevationally 1.2 cm away from the probe center), but also acts as a coupling medium for ultrasonic waves.

In the stationary scanning mode, the volunteers’ forearms were immersed in a water tank and scanned by the same imaging probe. The imaging probe was fixed on a motorized stage and moved at 140 mm in elevation direction at a constant speed of 2.5 mm/sec. In both scanning modes, the laser excitation wavelength was set to 850 nm. All volunteers and experimenters wore laser safety glasses to prevent eye damage from accidental laser irradiation.

## 3 Result

### 3.1 Processing time and frame rate

To compare the computing performance, the beamforming processing times of the DAS, DAS-CF, DMAS, and proposed DMAS-CF beamforming methods were measured on a desktop computer with a 64-bit CPU (i7-4790, Intel, USA), 24GB RAM and a GPU board (GeForce GTX 970, NVIDIA, USA). We used RF data acquired from the programmable clinical US/PA imaging system with an imaging depth of 4 cm. In this measurement, the beamforming process was accelerated through GPU processing with CUDA and the time to load RF data from hard disk to GPU memory was ignored.

Table 1 is a summary of the result. In the DAS beamforming, it took 7.5 ms and 7.6 ms to reconstruct a B-mode image without CF and with CF, respectively. The processing times using the DMAS were only about 1.5 times slower than DAS and is independent of CF*.*

**Table 1:** The measured processing time of DAS^a^*vs*. DMAS^b^ on PC. (unit: ms/Bscan).

	DAS	DMAS
**w/o CF**^c^	7.5 (× 1.0)	11.1 (× 1.5)
**w/ CF**	7.6 (× 1.0)	11.3 (× 1.5)

a Delay-and-sum.

b Delay-multiply-and-sum.

c Coherence factor.

We implemented the all the beamforming codes into the programmable clinical PA/US system with an integrated GPU board (GeForce GTX 1080, NVIDIA, USA) for real-time *in vivo* clinical evaluation. We scanned the human arms with DAS-CF and DMAS-CF beamformers and observed that the frame rates were not affected by the algorithm used, which demonstrates the real-time imaging capability of the proposed DMAS-CF algorithm.

### 3.2 Phantom imaging

**Fig. 3:**
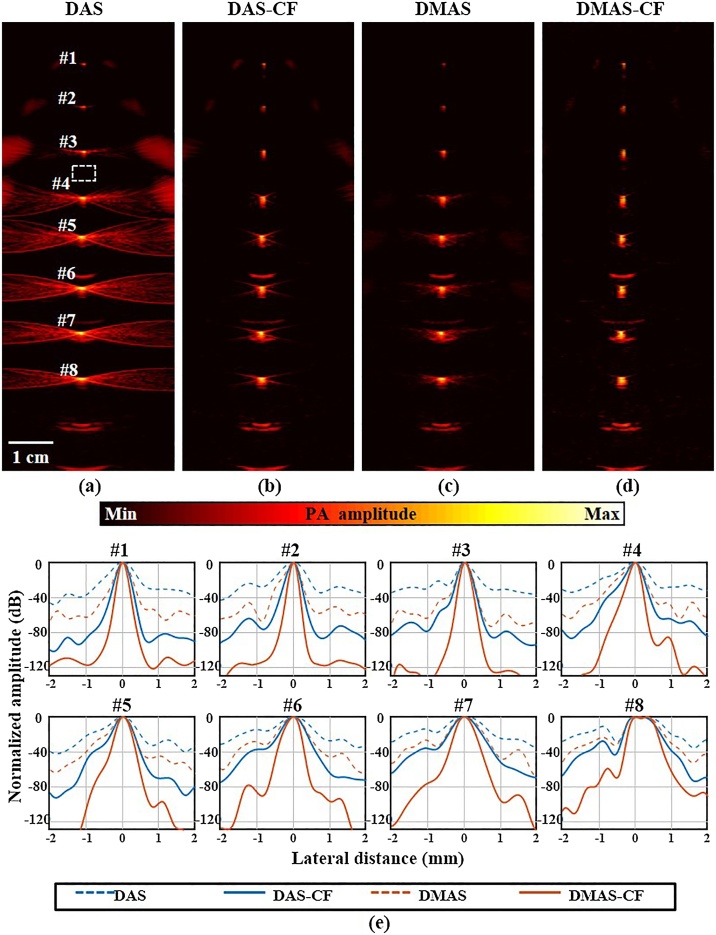
(a–d) PA phantom images reconstructed by DAS, DAS-CF, DMAS, and DMAS-CF beamforming algorithms, respectively. (e) Normalized lateral line profiles of the line targets highlighted with the white texts #1 - #8 in.(a–d). PA, photoacoustic; DAS, delay-and-sum; DMAS, delay-multiply-and-sum; and CF, coherence factor.

We acquired RF data of the nylon thread phantom using the clinical PA/US imaging system and reconstructed the PA images with all the beamforming methods. Figs. 3a and 3b are the DAS images without and with CF, respectively, and Fig. 3c and 3d are the DMAS images without CF and with CF (proposed), respectively. Note that signals are normalized by the aperture size for each depth and dynamic range of each image was set from 0.80*β* dB to 0 dB, where *β* is the average value of each background area highlighted with the white box in Fig. 3(a). Note also that we did not average any image. In DAS, we observed the strong grating lobes and side lobes next to the targets #1 - #4 and #3 - #8, respectively. However, the side and grating lobes are significantly reduced in DAS-CF and DMAS, and almost disappeared in DMAS-CF*.* This is because the main lobe combines signals of the same phase, but the grating lobe combines signals with a phase difference that is an integer ( ≠ 0) multiple of the carrier wavelength. The fractional waveform of the PA signal varies with the period, and thus, the combined signals in the grating lobe have relatively low coherence when compared to the main lobe. We observed some artifacts below the target #5 and #8, which are signals reflected from the bottom of the water tank.

We extracted line profiles from each thread in the lateral direction and measured the corresponding full width at half maximums (FWHMs) (Fig. 3e) to determine the lateral resolution. We also calculated the SNRs of each target as the ratio between the peak signal and the standard deviation of the background noise. To calculate the SNRs, the noise regions were selected as the signal present at the same depth as of peak but more than 6 mm away in the lateral direction from each peak.

The calculated FWHMs and the improvements for each beamformer are summarized in Table 2. The DAS’s FWHM is used as the standard to show the FWHM improvement rate of other algorithms. We calculated the geometric mean of FWHM improvement rates to quantify the resolution enhancement of each beamformer. The geometric means of FWHM improvement rate (*i.e.*, reduction rates) were 39.4%, 30.4% and 55.4% for DAS-CF, DMAS, and DMAS-CF, respectively, when compared to the FWHMs of DAS. Table 3 shows the SNRs and the corresponding improvement in comparison to DAS. The arithmetic means of the SNR improvement were 37.5 dB, 23.1 dB, and 93.6 dB in DAS-CF, DMAS, and DMAS-CF, respectively. Since the SNR unit is dB, the SNR improvement rates of each beamformer are expressed as the arithmetic means of the SNR increment.

**Table 2:** The measured FWHM^a^ and the Improvement Rate in the Phantom Images (Unit: μm).

	DAS		DAS-CF		DMAS		DMAS-CF (proposed)	
**#1**	452	0%	220	51.3%	259	42.7%	149	67.0%
**#2**	488	0%	211	56.8%	244	50.0%	134	72.5%
**#3**	342	0%	229	33.0%	253	26.0%	164	52.0%
**#4**	521	0%	280	46.3%	330	36.7%	191	63.3%
**#5**	512	0%	319	37.7%	354	30.9%	241	52.9%
**#6**	646	0%	393	39.2%	458	29.1%	262	59.4%
**#7**	774	0%	464	40.1%	533	31.1%	310	59.9%
**#8**	854	0%	664	22.2%	744	12.9%	604	29.3%
**G.M.**^b^		0%		39.4%		30.4%		55.4%

a Full width at half maximum.

b Geometric mean.

**Table 3:** The measured SNR^a^ and the Improvement Rate in the Phantom Images (Unit: dB).

	DAS		DAS-CF		DMAS		DMAS-CF (proposed)	
**#1**	40.0	0	64.6	24.6	60.6	20.5	109.4	69.4
**#2**	45.1	0	86.7	41.6	69.2	24.1	137.6	92.5
**#3**	46.0	0	96.0	50.0	74.0	28.0	153.8	107.8
**#4**	44.7	0	88.7	44.0	69.4	24.7	148.7	104.0
**#5**	45.8	0	87.7	41.9	68.2	22.4	148.2	102.4
**#6**	44.5	0	79.5	34.9	67.1	22.5	137.0	92.5
**#7**	44.8	0	75.8	31.0	66.5	21.7	139.3	94.5
**#8**	43.7	0	75.7	32.0	64.8	21.1	129.5	85.8
**A.M.**		0		37.5		23.1		93.6

a Signal-to-noise ratio, ^b^Arithmetic mean.

### 3.3 *In vivo* human imaging

In the handheld scanning mode, we imaged the forearm while gently moving the probe to demonstrate real-time imaging capability of our system (Supplementary video 1). Note that the underlying gray-color images are US B-mode. Both US images were reconstructed with DAS regardless of the PA reconstruction algorithm. The quality of the displayed B-mode images such as the spatial resolution and SNR varied significantly depending on the beamforming method employed. Frame rate, however, as discussed before, did not change significantly.

**Fig. 4:**
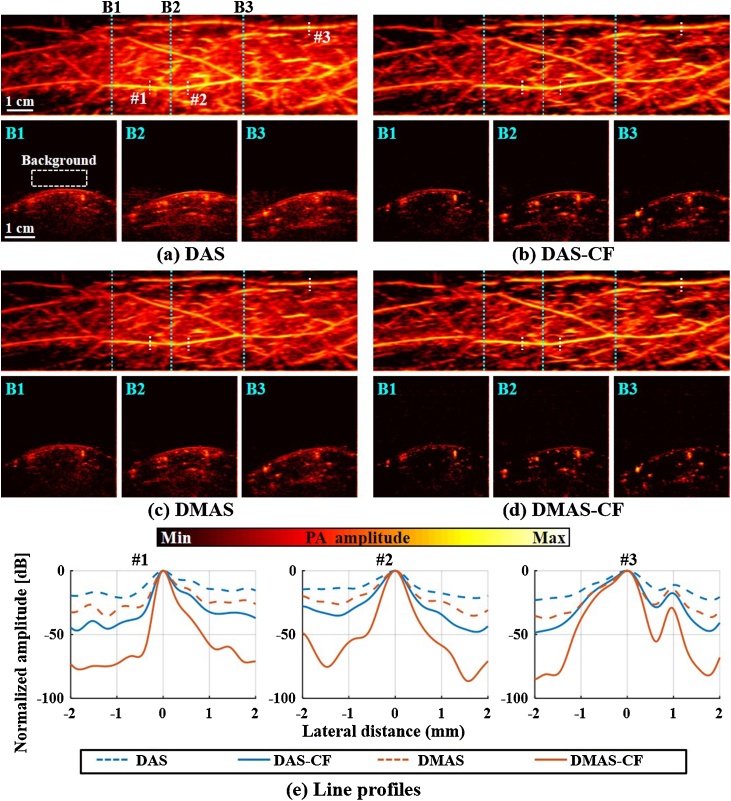
(a–d) PA MAP images (top) of a human forearm and the B-mode images (bottom) from the dashed regions in the MAP images. The images were reconstructed by DAS (a), DAS-CF (b), DMAS (c), and DMAS-CF (d) beamforming algorithms. (e) Normalized lateral line profiles of the targets highlighted with the white texts #1, #2, and #3 in.(d). PA, photoacoustic; MAP, maximum amplitude projection; DAS, delay-and-sum; DMAS, delay-multiply-and-sum; and CF, coherence factor.

The image quality for all beamforming methods was assessed quantitatively with the PA images acquired in the stationary scanning mode. Fig. 4a shows a representative maximum amplitude projection (MAP) and B-mode PA images of a human forearm reconstructed with DAS, DAS-CF, DMAS, and DMAS-CF algorithms in the stationary scanning mode. We then adjusted the dynamic range of the MAP and B-mode images to be from 0.65*β* dB to 0 dB and from 0.85*β* dB to 0 dB, respectively, where *β* is the average value of each background area highlighted with the white box in Fig. 4a. Similar to the phantom imaging result, the DAS had strong side lobes so it was difficult to distinguish the blood vessels in both MAP and B-mode images (Fig. 4a). In DAS-CF (Fig. 4b) and DMAS (Fig. 4c) images, we observed that the side lobes were relatively suppressed compared to the DAS image. The proposed DMAS-CF showed the weakest side lobes (Fig. 4d). To measure the FWHMs, we selected three peaks (marked with white text #1, #2, and #3 in Fig. 4d) in the PA MAP images and extracted their line profiles in the azimuth direction (Fig. 4e). Table 4 shows the measured FWHMs and the improvement rates compared to DAS. The geometric means of FWHM improvement rates were 38.7%, 36.4% and 54.5% in DAS-CF, DMAS, and DMAS-CF compared to DAS, respectively. We also measured the SNRs of each peak and calculated their improvement rates compared to DAS (Table 5). When calculating the SNRs, the highlighted region with the white dashed boxes in Fig. 4a was used as the noise area. DAS-CF, DMAS, and DMAS-CF showed the SNR improvement rates of 21.9 dB, 5.9 dB, and 47.4 dB, respectively, compared to DAS.

**Table 4:** Measured FWHM^a^ and the Improvement Rate in the Human Forearm Images (Unit: μm).

	DAS		DAS-CF		DMAS		DMAS-CF (proposed)	
**#1**	532	0.0%	283	46.8%	320	39.8%	209	60.7%
**#2**	623	0.0%	392	37.1%	437	29.9%	289	53.6%
**#3**	729	0.0%	486	33.3%	434	40.5%	366	49.8%
**G.M.**^b^		0.0%		38.7%		36.4%		54.5%

a Full width at half maximum.

b Geometric mean.

**Table 5:** Measured SNR^a^ and the Increment in the Human Forearm Images (Unit: dB).

	DAS		DAS-CF		DMAS		DMAS-CF (proposed)	
**#1**	43.5	0.0	70.0	26.5	52.4	8.9	102.5	58.9
**#2**	40.1	0.0	64.3	24.2	48.3	8.2	89.0	48.8
**#3**	41.0	0.0	56.2	15.2	41.5	0.5	75.4	34.4
**A.M.**^b^		0.0		21.9		5.9		47.4

a Signal-to-noise ratio.

b Arithmetic mean.

## 4 Discussion and conclusion

**Fig. 5:**
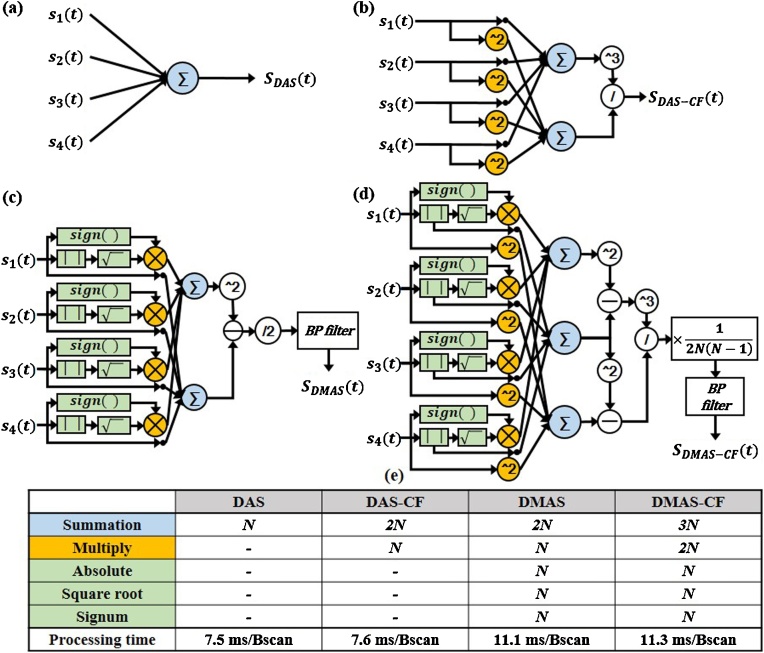
(a–d) Diagrams of DAS, DAS-CF, DMAS, and DMAS-CF beamforming methods, respectively. (e) The approximate number of operations and the total processing time required for each beamforming method.

In this work, we introduced an enhanced DMAS-CF algorithm, which uses the coherence of received signals to suppress side lobes and noise and thus improve lateral resolution and SNR in PA imaging. We also optimized this algorithm for real-time implementation into the clinical system. For a quantitative assessment of image quality as well as computing performance, we implemented the DAS and DMAS beamformers with/without CF on both a PC and a programmable US/PA system. Fig. 5 summarizes the processing flow, number of operations, and total processing time for each beamforming method. The proposed DMAS-CF only requires 0.2 ms/Bscan longer processing time than DMAS, even though it had almost twice as many multiplications as DMAS. Similarly, DAS-CF has *N* more multiplications than DAS, but the processing time was only 0.1 ms/Bscan longer. Thus, we can infer that the number of multiplication operations in the proposed DMAS-CF algorithm is reduced enough not to affect the processing speed. Since DAS only has *N* summations, but it still took 7.5 ms/Bscan, we can infer that the processing time in DAS is mainly consumed by the memory accesses to load and store data. DMAS, on the other hand, has additional signum, absolute, and square root operations as compared to DAS-CF and was approximately 3.5 ms/Bscan slower. From the above, we can deduce that three operations for the DMAS resulted in the slowdown in DMAS computation since additional computations required in DMAS (multiplications and summations) were hidden behind the memory access times. Therefore, the processing time in the proposed DMAS-CF beamforming algorithm was mainly increased due to the basic memory access procedure (about 7.5 ms/Bscan) and the additional signum, absolute, and square root operations (about 3.5 ms/Bscan).

The proposed DMAS-CF is about 50% slower than the DAS, but it is still fast enough for real-time imaging in simulation in our system. In this study, we used a laser system with a PRF of 10 Hz and a clinical PA/US imaging system with 64 channels, which was half the number of the transducer elements. Thus, to generate one frame, two laser shots and two ultrasonic reception are necessary, fixing the framerate at 5 fps. Therefore, if a 128-channel clinical imaging system was used, the maximum speed of the laser PRF could be available. Recently, several PA imaging systems using a light source with a high PRF have been developed such as laser diodes [37] and light emitting diodes (LEDs) [14,38]. Therefore, the proposed DMAS-CF will be useful for such high-speed PA imaging systems.

FWHM and SNR are best in DMAS-CF, followed by DAS-CF, DMAS, and DAS, regardless of depth. The resolution and SNR improvements of DMAS are known to be due to the doubled center frequency and the increased effective aperture after the combinatorial multiplication [24]. Meanwhile, CF improved the image quality by suppressing the side lobes and background noise according to the ratio of the DC energy, associated with the signal coherence, to the total energy of the synthesized signals [39]. By simultaneously exploiting the inherent advantages of DMAS and CF, DMAS-CF was able to further suppress the side lobes and noise levels. Additionally, the CF of the DMAS-CF was designed to calculate the coherence of the received signals extended from *N* to *N(N-1)/2*, having a weight from 0 to 1 like the CF [31] and the generalized CF [39]. Thus it was able to restrain the side lobes and noise levels more robustly than in DAS-CF*.* This resulted in the best lateral resolution and SNR in the phantom and *in vivo* studies.

In conclusion, we developed a novel beamformer that combines DMAS and CF and demonstrated real-time PA imaging on the clinical US/PA imaging system. The proposed beamformer is fast enough to enable real-time imaging and provides improved image quality enhancement compared to DMAS and DAS-CF*.* Therefore, we believe that this proposed beamforming method could be integrated into any clinical PA device for improved image quality for better clinical outcome.

## Declaration of Competing Interest

Chulhong Kim and Kijong Lee have financial interests in OPTICHO, which, however, did not support this work.
